# Transcriptomic Study of Diffuse Large B-Cell Lymphoma Associated with HIV Infection: Identification of Novel Molecular Subtypes

**DOI:** 10.32604/or.2026.076241

**Published:** 2026-07-16

**Authors:** Yasmine Labiad, Céline Baier, Michèle Genin, Caroline Besson, Sophie Prevot, Hubert Lepidi, Régis Costello

**Affiliations:** 1 Aix Marseille Univ, TAGC/INSERM UMR1090, Parc Scientifique de Luminy, Marseille, France; 2 Institut Pierre Louis d’Epidémiologie et de Santé Publique (IPLESP) UMRS1136 INSERM et UPMC, Paris, France; 3 Service d’Hématologie Oncologie, Centre Hospitalier de Versailles, Versailles, France; 4 Centre de Recherche en Epidémiologie et Santé des Populations (CESP), INSERM U1018, Université Paris-Saclay, Villejuif, France; 5 Service d’Anatomopathologie, AP-HP, Le Kremlin-Bicêtre, France; 6 Service d’Anatomopathologie, AP-HM, CHU La Timone, Marseille, France; 7 Service d’Hématologie et Thérapie Cellulaire, AP-HM, CHU La Conception, Marseille, France

**Keywords:** Diffuse large B-cell lymphoma, HIV-associated lymphoma, transcriptomic profiling, molecular subtypes, gene expression analysis, biomarker discovery

## Abstract

**Objectives:** Transcriptomic profiling has enabled the classification of Diffuse Large B-Cell Lymphoma (DLBCL) into distinct subtypes, such as Germinal Center B-cell-like (GCB) and Activated B-cell-like (ABC), primarily in HIV-negative patients. However, HIV-associated DLBCL may follow different molecular mechanisms due to immune dysregulation. This study aimed to characterize the transcriptomic landscape of HIV-related DLBCL to identify distinct subtypes and deregulated pathways with potential theranostic implications. **Methods:** Twelve formalin-fixed, paraffin-embedded DLBCL samples from HIV-positive patients were analyzed using Agilent’s microarray. Quantile normalization and unsupervised hierarchical clustering were performed to classify tumors based on gene expression profiles. **Results:** Two distinct transcriptomic subgroups were identified. *TP53* and *BCL7A* were overexpressed in cluster I, while *BCL2* was overexpressed in cluster II. Notably, the “immune system development” pathway was under expressed in cluster I compared to cluster II. **Conclusions:** Our findings reveal two molecularly distinct subtypes of HIV-associated DLBCL, likely driven by differences in tumor microenvironment and immune status. These transcriptomic profiles may guide future targeted therapies. Further validation in larger cohorts and integration with proteomic and clinical data are warranted to develop a comprehensive theranostic framework.

## 1 Introduction

Diffuse large B-cell lymphomas (DLBCL) represent 30–40% of lymphomas and are treated with a combination of chemotherapy and immunotherapy drugs. The International Prognostic Index (IPI), used to classify and treat patients, is calculated on the basis of clinical parameters: age, LDH level, anatomical stage, extra-nodal localizations, and performance status, but recent publication suggest the NCCN-IPI should be considered as the reference model [1]. In a seminal paper, Alizadeh et al. [2] used microarrays analysis of tumor biopsies from patients with DLBCLs to propose the distinction of two different subtypes. These categories remain the basic elements of the World Health Organisation’s 2022 classification of DLCLs [3]. The GC (germinal center) subtype is defined by the overexpression of CD10, CD38, A-myb, OGG1, BCL-6, BCL-7A, and LMO2, while the ABC (activated B cell) subtype is characterized by the overexpression of IRF4, FLIP, and BCL-2, with its proliferation seemingly relying on the NF-κB pathway. The GC lymphomas are considered to have a better prognosis than the ABC type. Based on these molecular profiles, attempts have been made to specifically target the metabolisms pathways used in each subtype [4]. Moreover, attempts to use a simpler technique for its use in routine practice have been proposed, such as the immunochemistry algorithm of Hans et al. [5] or Muris et al. [6], which strict correlation with transcriptomic data is not observed. Since ABC-DLBCL proliferation and survival rely on BCR-dependent NF-kB signaling, inhibitors of Bruton tyrosine kinase (BTK) could be useful, although more trials are required before the validation for routine treatment [7,8]. Additional subgroups have further been identified, since a 17-gene model allowed to divide DLBCL in quartiles with five-year survival ranging from 15% to 73% [9]. These profiles correspond to different prognostic groups, at least in relapsed/refractory patients [10] although this issue is debated [11] and probably depends on the technical approach used, transcriptomic profiling versus various algorithms [12]. Recently, the Hans-based cell of origin has been severely criticized for its poor prognostic significance [13].

Notably, these studies were performed in HIV-negative patients, although non-Hodgkin’s lymphomas in HIV-infected patients include specific pathological forms (serous lymphomas, multicentric Castleman’s disease), in addition to histological forms found in non-immunocompromised patients, including Burkitt’s lymphoma and DLBCL [14]. Although this recent WHO classification is no more exclusively based on the lymphoma disease background (HIV-related, post-transplantation, primary immunodeficiency, iatrogenic immunodeficiencies), HIV infection has some specificity [15]. The presence of HIV induces a particular microenvironment in the lymph node that induces, among other effects, activation and proliferation of B cells in the absence of the immune response. This proliferation increases the deregulation of genes such as p53 and the activation of proto-oncogenes such as c-myc and BCL-6 in addition to the absence of immune surveillance. Moreover, the immunosuppression linked to CD4 T-cell depletion allows the proliferation of EBV and of KSHV/HHV8 viruses which encoded proteins stimulate B cell proliferation and thus lymphomagenesis [16]. Nonetheless, HIV itself could have a direct impact via the expression of HIV p17 protein variants that accumulate in lymph node (even in the absence of HIV detection) and are able to activate the PI3K/AKT signaling pathway [17]. These HIV p17 protein variants share insertions in their C-terminal region that modify their biologic properties. Although recent advances in anti-retroviral treatment allow most patients with controlled HIV infection to be treated like the general population [18], due to these particularities of HIV-related lymphomas, the existence of the two GEP signatures GC vs. ABC in HIV-related lymphomas was not evident. Thapa et al. [19] have focused their study on the expression of microRNAs, since these molecules have been shown to play a significant role more specifically in EBV-related tumorigenesis [20]. In this study they have shown that the mi-17-92 paralog clusters were upregulated in B cells more particularly at the GC stage, in the eight analyzed DLBCL but also in three other subtypes of HIV-related lymphomas (Burkitt, central nervous system, primary effusion lymphoma) [19]. The study of Ramos et al. [21] analyzed the expression of NF-kB target genes in HIV-related lymphomas. They observed the expression of tissue origin-specific markers in PEL (CD69, CSF-1, CIQBP), of IL1beta, cyclin D3 and CD48 in KS, and identified CCR5 as a key marker in Burkitt lymphoma [21]. To differentiate HIV-related from the other DLBCL, Capello et al., and Rinaldi et al. performed a genome-wide DNA profiling [22,23]. They concluded that HIV-related had specific genetic lesions since fragile sites-associated genes were more frequently inactivated, more particularly *FHIT* (FRA3B), *WWOX* (FRA16D), *DCC* (FRA18B) and *PARK2* (FRA6E), in comparison with non-HIV related DLBCL. In the study of Chapman et al. [24] thirty HIV-related DLBCL were analyzed. Interestingly the HIV-related lymphomas had more frequent MYC rearrangements or mutations than non-HIV-related lymphomas, and in contrast had rarely BCL2 rearrangements. The authors used the Hans algorithm [5] to classify these HIV-related lymphomas and concluded that half of the sample used were of GC lymphoma type, but the other half could not be classified as ABC. These results were confirmed by cell of origin (COO) LymphGen tool [25]. Of note, the percentage of lymphomas classified as other than GC but not ABC (50%) is quite high in comparison with the results usually obtained in non-HIV related lymphomas, i.e., ≈50% of ABC types [2]. The very interesting study of Madan et al. [26] analyzed by immunohistochemical staining of tissue microarrays the expression of GC markers (BCL6, CD10, CyclinH) vs. ABC markers (MUM1, CD138, PAK1, CD44, BCL2) in 12 HIV-related and 27 non-HIV related DLBCL. The immunostaining, as expected, clearly identified two distinct clusters of GC and ABC types in non-HIV related DLBCL, while in the case of HIV-related lymphomas only a single aggregate was identified, that moreover expressed an intermediate GG/ABC phenotype [26]. Interestingly, the study of Patrone et al. [27] allows some different conclusions in comparison with the previous studies. They analyzed subtractive hybridization (SSH) to isolate differentially expressed genes from HIV-related and non-related DLBCL. They progressively restricted the study from 1800 to 18 candidate genes. Unfortunately, there was no preferential expression of these genes in HIV-related vs. non-HIV-related lymphomas. This study had nonetheless significant limitations, i.e., the number of analyzed samples and the restriction of analyzed genes to those already annotated in data banks [27].

HIV-associated diffuse large B-cell lymphoma (DLBCL) differs significantly from non-HIV-associated DLBCL in both clinical and molecular features. In HIV-positive patients, chronic immune activation and CD4 T-cell depletion create an immunosuppressive environment that promotes the proliferation of oncogenic viruses such as Epstein-Barr virus (EBV) and Kaposi’s sarcoma-associated herpesvirus (KSHV/HHV8), which contribute to lymphomagenesis through the expression of viral proteins like LMP1 and v-FLIP. Molecularly, HIV-associated DLBCL often exhibits higher rates of MYC rearrangements and reduced incidence of BCL2 alterations compared to non-HIV DLBCL, indicating alternative oncogenic pathways in its development. Furthermore, gene expression profiling studies reveal a higher prevalence of unclassifiable or intermediate phenotypes, blurring the distinction between the germinal center (GC) and activated B-cell (ABC) subtypes that are typically observed in immunocompetent patients.

Finally, the validation of the GC/ABC subtypes in HIV-related lymphomas must be confirmed. For these reasons, the objective of our study was to analyze the gene expression profile of DLBCL to verify the possible existence of subgroups described in immunocompetent patients and/or to define new gene profiles specific to HIV-infected non-Hodgkin’s lymphoma (NHL). Our goal was also to define the pathophysiology of the different subtypes and to identify deregulated molecular pathways that may have prognostic value.

## 2 Materials and Methods

### 2.1 Biological Samples

Seven formalin-fixed paraffin-embedded (FFPE) tumor samples, together with associated clinical and biological data, were obtained from the ANRS (Agence Nationale de Recherches sur le Sida et les hépatites virales) CO16 LYMPHOVIR cohort. The cohort was conducted within the framework of an ANRS-sponsored research program and in accordance with French regulatory and ethical requirements. The corresponding database received authorization from the French Data Protection Authority (CNIL) on 28 May 2007 (Inserm epidemiological database ID: 60062). Ethical oversight for the cohort was provided by the Comité de protection des personnes Île-de-France VII.

Five additional FFPE samples were provided by Pr. H. Lepidi (CHU La Timone, Marseille) and Pr. N. Mounier (CHU de Nice), with approval obtained from the corresponding local ethics committees (Comité de protection des personnes Sud-Méditerranée II and Comité de protection des personnes Sud-Méditerranée V).

All biological samples were initially collected as part of routine diagnostic and medical care procedures and were subsequently used for research purposes in accordance with institutional regulations and the Declaration of Helsinki. Patients provided informed consent and/or non-opposition for the use of their biological samples and associated clinical data for research purposes and publication. Due to the historical nature of the cohort and archival procedures, specific public registration numbers for certain local approvals are no longer available in the institutional records.

Two technical replicates were used for each biological sample to ensure the reliability and reproducibility of the transcriptomic analyses. The clinical characteristics of the patients are summarized in Table 1.

**Table 1:** Clinical and Immunophenotypic characteristics of diffuse large B-cell lymphoma samples from HIV-infected patients.

Samples	Age at Inclusion, Years	Sex	Histological Grade	Infectious Statut	Immunophenotyping
C075-2A	47	Male	Diffuse large B-cell lymphoma	HIV^+^	CD20^+^, CD10^−^, CD138^−^, BCL2^+^
C053-A	50	Male	Diffuse large B-cell lymphoma	HIV^+^	CD20^+^, CD10^+^, BCL6^+^, CD138^−^
C066-B1	38	Male	Diffuse large B-cell lymphoma	HIV^+^	CD20^+^, CD138^+^, BCL2^+^, CD30^−^, ALK1^−^
C067-A	45	Male	Diffuse large B-cell lymphoma	HIV^+^	CD20^+^, CD10^−^, BCL6^−^
C135-2	34	Female	Diffuse large B-cell lymphoma	HIV^+^	CD5^+^, CD20^+^, CD10^+^, BCL6^+^, BCL^−^
C126-C	81	Male	Diffuse large B-cell lymphoma	HIV^+^	CD5^−^, CD20^+^, CD10^−^, BCL6^+^, BCL2^+^
C071-A1	67	Male	Diffuse large B-cell lymphoma	HIV^+^	CD71^−^, CD20^+^, CD10^−^, BCL6^−^
183644-LB4	39	Male	Centroblastic Diffuse large B-cell lymphoma	HIV^+^	CD45^+^, CD20^+^, CD79A^+^, CD3^−^, CD30^−^, ALK1^−^
190078-LB3	39	Male	Diffuse large B-cell lymphoma	HIV^+^, HCV^+^	CD45^+^, CD138^+^, CD20^−^, CD3^−^, CD56^−^, CD5^−^, CD8, CD79A^−^
10H4867-LB2	45	Male	Centroblastic Diffuse large B-cell lymphoma	HIV^+^, HCV^+^	CD20^+^
154575-LB3	NA	NA	Diffuse large B-cell lymphoma	HIV^+^	CD20^+^
126221-LB3	NA	NA	Diffuse large B-cell lymphoma	HIV^+^	CD20^+^

Abbreviations: HIV, human immunodeficiency virus, HCV: hepatitis C virus, CD: cluster of differentiation, ALK: Anaplastic Lymphoma Kinase, BCL: B-cell lymphoma. NA: not available due to age/sex missing to archival issues.

### 2.2 RNA Extraction, Quantification, and Quality Control

RNA was extracted using the RNeasy FFPE kit from Qiagen France (Courtaboeuf, France, catalog number 73504). The process began with deparaffinization, achieved by immersing the samples in xylene to remove paraffin, followed by ethanol washes to rehydrate the tissue. After rehydration, the tissue underwent lysis with proteinase K at 56°C for 15 min, followed by a heat-induced crosslink reversal step at 80°C for 15 min. This step ensures the recovery of high-quality RNA suitable for downstream analysis. RNA was then purified using on-column DNase treatment to eliminate any contaminating DNA, followed by several washing steps to remove impurities. RNA was eluted in RNase-free water and quantified using a NanoDrop spectrophotometer 2000 (Thermo Fisher Scientific, Santa Clara, CA, USA). The integrity of RNA was assessed using the Agilent Bioanalyzer 2100 (Agilent Technologies, Santa Clara, CA, USA). Samples exhibiting a RNA integrity number (RIN) ≥ 3 were considered of sufficient quality for transcriptomic analysis. Samples with suboptimal RIN scores were excluded from further processing.

### 2.3 Transcriptomic Study

For transcriptomic analysis, Agilent SurePrint G3 Human GE 8 × 60K v2 chip was used (Agilent SurePrint G3 Human Gene Expression 8 × 60K v2 Microarray Kit (Agilent Technologies, Part No. G4851B, Design ID 039494). The chip provides high genes and transcript coverage with high sensitivity. The chip has eight arrays, each with 62976 probes. Sample preparation, labeling, and hybridization were performed according to the Agilent Gene Expression FFPE Workflow protocol (PDF Link: https://www.manuallib.com/download/pdf11/AGILENT-GENE-EXPRESSION-FFPE-WORKFLOW-QUICK-START-GUIDE.PDF). The protocol was optimized for formalin-fixed, paraffin-embedded (FFPE) samples to ensure accurate and reproducible data. The Gene Expression FFPE Workflow protocol by Agilent is a specialized kit designed to address the unique challenges associated with formalin-fixed, paraffin-embedded samples. FFPE tissues. This protocol is specifically optimized to handle degraded RNA, ensuring that meaningful transcriptomic data can still be obtained. By addressing the inherent challenges of FFPE samples, this workflow allows us to leverage valuable archival specimens for robust and reliable transcriptomic studies.

The FFPE workflow begins with an RNA input repair step to address RNA fragmentation caused by the fixation process. RNA is reverse-transcribed into complementary DNA (cDNA) using a T7-oligo(dT) primer. This cDNA is amplified by *in vitro* transcription with T7 RNA polymerase, generating amplified complementary RNA (aRNA). The aRNA is then fluorescently labeled with Cy3 dye, purified to remove unincorporated dye, and quantified to ensure labeling efficiency. Hybridization of labeled aRNA to the microarray is performed under stringent conditions at 65°C for 17 h to ensure specific binding of probes to their target sequences. Arrays are washed to remove non-specifically bound material, scanned using the Agilent G4900DA SureScan Microarray Scanner (Agilent Technologies, Inc., Santa Clara, CA, USA), and fluorescence intensities are extracted using Agilent Feature Extraction software. The raw transcriptomic data generated and analyzed in this study have been deposited in the BioStudies database, under the ArrayExpress collection. The dataset is publicly accessible under the accession number E-MTAB-15172.

### 2.4 Statistical Analysis

The AgiND library, implemented in the R software environment, was utilized for data analysis and visualization. AgiND, based on the Bioconductor framework, provides robust diagnostic tools for assessing microarray data quality and normalization. To ensure consistency across samples, quantile normalization was employed, homogenizing intensity distributions to minimize technical variations. The AgiND library used in this study was version 1.12.0, compatible with R version 4.2.2. Two filters were applied to preprocess the raw data: (1) control probes were removed to eliminate noise introduced by non-informative probes, and (2) genes expressed below the background level in 100% of samples were excluded to focus on biologically relevant signals. The background level was defined based on negative control probes included in the microarray platform.

For clustering, unsupervised hierarchical clustering was performed on normalized, median-adjusted gene expression data, grouping both genes and samples based on their expression profiles. The clustering analysis was carried out using the TMeV (Tigr MultiExperiment Viewer software, version 4.9.0), which is part of the TM4 Microarray Software Suite (http://www.tm4.org/mev/). Unsupervised classification relies on gene expression data, enabling the separation of patient samples into distinct groups with shared transcriptomic signatures. Pearson correlation was chosen as the similarity metric, and clustering was conducted using the average linkage method to compute inter-cluster distances. Adjustments in TMeV included applying the “Median Center Gene/Row” method for data centering and setting the color scale limits between −2 and 2 for visualization.

Subsequently, Significance Analysis of Microarrays (SAM) was performed within TMeV to identify differentially expressed genes between groups defined by unsupervised clustering. The SAM analysis was configured to run with 10,000 permutations, a false discovery rate (FDR) of 1%, and a minimum fold-change threshold of 2, ensuring high confidence in the results. Additionally, visualization of heatmaps and dendrograms was done directly within TMeV, applying the “Median Center Gene/Row” method for data centering and setting the color scale limits between −2 and 2 for visualization.

### 2.5 Gene Enrichment Analysis

Gene enrichment analysis was performed using g: Profiler (https://biit.cs.ut.ee/gprofiler/gost, g: Profiler version e111_eg58_p18_f463989d, database updated on 25/01/2024). This tool was used to identify enriched biological pathways, molecular functions, and cellular components associated with the differentially expressed genes from the microarray analysis. The gene set for the analysis was derived from the filtered microarray data, where genes with an FDR (false discovery rate) of 1% and a fold-change of 2 were considered differentially expressed. The analysis was conducted using default settings, and Ensembl genome database (version 104) was chosen as the reference background to map the genes and account for any gene name inconsistencies. For multiple testing, g: Profiler’s g: SCS (generalized Smith-Waterman-Cost-sensitive) algorithm was used to adjust for multiple comparisons, reducing the likelihood of false positives. This adjustment ensures that the reported pathways, molecular functions, and cellular components are significantly enriched. The enrichment analysis specifically focused on Gene Ontology (GO) terms, which were categorized into biological processes, molecular functions, and cellular components. The adjusted *p*-value threshold for significance was set at 0.05, ensuring that only highly reliable and biologically meaningful pathways were considered. Additionally, the Benjamini-Hochberg correction was applied for controlling the false discovery rate (FDR), ensuring the robustness of the results in the context of multiple hypothesis testing.

## 3 Results

**Figure 1:**
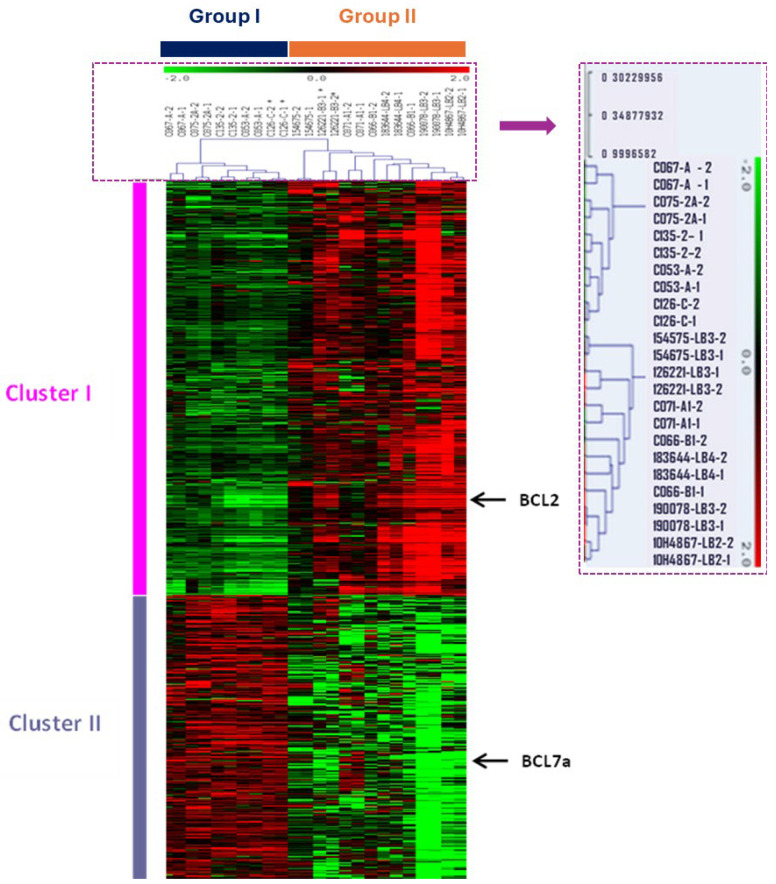
Transcriptomic profiles of HIV-associated diffuse large B-cell lymphomas (DLBCL). Heatmap representing the differential gene expression profiles of 24 samples (12 biological samples with two technical replicates each). The samples are divided into two distinct transcriptomic subgroups (Cluster I and Cluster II) based on unsupervised hierarchical clustering. Genes with significantly altered expression levels are displayed on the ordinate axis, with overexpressed genes shown in red and under expressed genes in green. The abscissa separates the samples into the identified clusters, highlighting distinct gene expression patterns between the groups. The clustering was performed using normalized data (quantile normalization) and median-adjusted gene expression levels, with Pearson correlation as the similarity metric and average linkage as the clustering method. These transcriptomic signatures underline the heterogeneity of HIV-associated DLBCL and suggest potential molecular differences between the two clusters. A color-blind reader version of the figure is available as Supplementary Materials.

After hierarchical clustering of the normalized data of the 24 samples (2 replicates per sample), two groups were highlighted according to their gene expression profile. Based on this, a SAM analysis was performed on the normalized data using Pearson correlation and 10,000 permutations, with the False discovery rate (FDR) set to 1% and a fold change of 2. The two groups identified by the initial unsupervised hierarchical clustering were confirmed, characterized by the differential expression of 4045 genes (Fig. 1, a color-blind reader version of the figure is available as Supplementary Materials).

These two clearly defined subgroups did not correspond to the GC versus non-GC transcriptomic subgroups, except for the expression of the BCL2 and BCL7A genes. Among the most significant differences, the BCL2 gene was overexpressed in patient group II; specifically, BCL2 exhibited a 2.61 log_2_ fold change (adjusted *p*-value < 0.01) when comparing group II to group I corresponding to a fold change of approximately +6.07. While BCL7A was over-expressed in patients’ group I; specifically, BCL7a exhibited a 2.69 log_2_ fold change (adjusted *p*-value < 0.01) when comparing group I to group II corresponding to a fold change of approximately +6.69.

In contrast with the transcriptomic classification of non-HIV related DLBCL, we failed to identify differential functional annotations specific to one subgroup. We were not able to define differential prognosis between the two groups due to the small number of samples analyzed. Functional annotation has enabled us to identify very general signaling pathways that are not directly related to pathology. The several signaling pathways identified are summarized in Table 2 and Table 3.

**Table 2:** Cluster I functional annotation. Cluster I genes are under-expressed in patients’ group I and over-expressed in patients’ group II.

Source	Term Name	Term ID	Adjusted *p* Value
GO:MF	Protein binding	GO:0005515	7.10 × 10^−22^
GO:MF	Oxydoreduction transmembrane transporter activity	GO:0015453	8.4 × 10^−3^
GO:MF	Interleukin-3 receptor activity	GO:0004912	2.89 × 10^−2^
GO:MF	NADH dehydrogenase activity	GO:0003954	4.02 × 10^−2^
GO:MF	Cell adhesion molecule binding	GO:0050839	4.02 × 10^−2^
GO:MF	SH3 domain binding	GO:0017124	4.68 × 10^−2^
GO:MF	Regulation of nitrogen compound metabolic process	GO:0051171	2.17 × 10^−9^
GO:MF	Cell death	GO:0008219	5.9 × 10^−9^
GO:MF	Cellular response to stress	GO:0033554	5.07 × 10^−7^
GO:MF	Regulation of cell population proliferation	GO:0042127	1.88 × 10^−5^
GO:MF	Immune system development	GO:0002520	2.90 × 10^−5^
GO:MF	Mitochondríon organization	GO:0007005	3.26 × 10^−5^
GO:MF	Cell population proliferation	GO:0008283	3.70 × 10^−5^
GO:MF	ATP synthesis coupled to electron transport	GO:0042773	4.13 × 10^−5^
GO:MF	Hemopoiesis	GO:0030097	4.16 × 10^−5^
GO:MF	Hematopoíetic or Iymphoid organ development	GO:0048534	5.76 × 10^−5^
GO:MF	Regulation of cell communication	GO:0010646	5.99 × 10^−5^
GO:MF	Regulation of myeloid cell differentiation	GO:0045637	1.89 × 10^−4^
GO:MF	Positive regulation of cell population proliferation	G0:0008284	2.23 × 10^−4^

MF: Molecular functions.

**Table 3:** Cluster II functional annotation. The cluster II genes are over-expressed genes in patients’ group I and under-expressed in patients’ group II.

Source	Term Name	Term ID	Adjusted *p* Value
GO:MF	Protein binding	GO:0005515	2.86 × 10^−14^
GO:MF	Response to stimulus	GO:0050896	2.15 × 10^−5^
GO:MF	Cell differentiation	GO:0030154	1.87 × 10^−4^
GO:MF	Multicellular organísm development	GO:0007275	3.15 × 10^−4^
GO:MF	Cell communication	GO:0007154	9.60 × 10^−4^
GO:MF	Cell-cell signaling	GO:0007267	3.57 × 10^−3^
GO:MF	Tumor necrosis factor superfamily cytokine production	GO:0071706	1.15 × 10^−2^
GO:MF	Cell morphogenesis	GO:0000902	2.01 × 10^−2^
GO:MF	Cell surface receptor signaling pathway	GO:0007166	2.91 × 10^−2^
GO:MF	Positive regulation of dendritic cell cytokine production	GO:0002732	3.06 × 10^−2^
GO:MF	Cell migration	GO:0016477	3.2 × 10^−3^

MF: Molecular functions.

Our findings indicate that HIV-related DLBCLs exhibit a distinct transcriptomic profile compared to HIV-negative DLBCLs. This distinct profile could be partly attributed to the different microenvironments, cytokine profiles, and immune responses in these different lymphoma populations. The overexpression of BCL2 and BCL7A aligns with the notion that these genes play a critical role in the pathogenesis of HIV-related lymphomas. The lack of clear GC versus ABC subtype differentiation suggests that the underlying biology of HIV-associated DLBCL may involve unique pathogenic mechanisms not present in immunocompetent individuals. 

**Figure 2:**
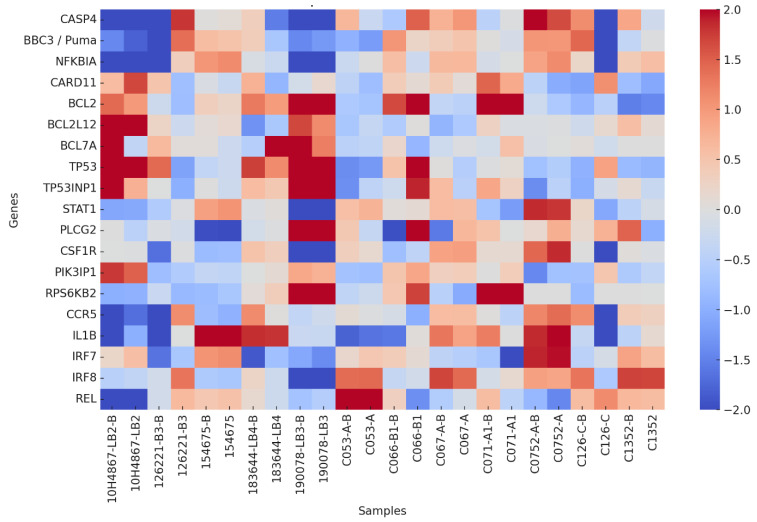
Heatmap Depicting the Expression of Differentially Expressed Genes Involved in Lymphoma Pathophysiology: The heatmap visualizes the expression patterns of a selection of genes differentially expressed in lymphoma and hematologic malignancies, focusing on pathways critical to their physiopathology. These include key genes implicated in cell proliferation (e.g., PI3K-AKT pathway, B-cell receptor pathway), apoptosis (e.g., BCL2, BCL2L12, BBC3/PUMA), inflammation and immune signaling (e.g., IL1B, STAT1, IRF7), and canonical pathways such as NF-κB and JAK-STAT. The expression levels are median centered to highlight relative upregulation (red) and downregulation (blue) across samples. Genes such as REL, BCL7A, and TP53 highlight their central role in HIV-associated lymphomas, while others like CARD11 and PLCG2 emphasize signaling disruptions in malignancies. A color-blind reader version of the figure is available as Supplementary Materials.

For example, the signaling pathway “immune system development “(adjusted *p*-value = 2.90 × 10^−5)^ identified in the cluster I functional annotation is under-expressed in patients’ group I and over-represented in patients’ group II. This pathway includes significative genes such as STAT1, BATF, IRF7, HLA-E and IL4-R, thus differentially expressed between the patients’ groups. STAT1 is a key transcription factor involved in interferon signaling pathways, crucial for antiviral responses and tumor surveillance. Dysregulation of STAT1 has been associated with immune evasion mechanisms in various lymphomas, including diffuse large B-cell lymphomas [28]. Other pivotal genes of the immune responses have also been identified. BATF is an important transcription factor that drives the differentiation of T cells and B cells. It is essential for the development of follicular helper T cells, which support antibody responses. BATF mutations or dysregulation can affect immune cell function and are implicated in lymphomagenesis [29]. The differential expression of immune-related genes such as *STAT1* and *BATF* points to the potential of immunomodulatory therapies aimed at restoring anti-tumor immunity. IRF7 is a transcription factor that regulates the production of type I interferons, critical for antiviral immunity and immune surveillance against tumors. It has been linked to immune evasion in lymphomas due to its role in regulating innate immunity [30]. HLA-E is involved in immune recognition and modulation, particularly through interactions with NK cells. Overexpression of HLA-E contributes to immune escape in lymphomas by inhibiting NK cell-mediated cytotoxicity [31]. IRF8 is another transcription factor implicated in the differentiation of myeloid cells and B cells. IL4R encodes the receptor for interleukin-4, a cytokine that promotes B cell proliferation and survival. A selection of genes that are differentially expressed in Patient Group I and II is summarized in Fig. 2 (a color-blind reader version of the figure is available as Supplementary Materials).

## 4 Discussion

DLBCLs represent a heterogeneous group of aggressive lymphoid malignancies characterized by diverse genetic alterations and distinct clinical behaviors. Among the differentially expressed genes, we identified TP53, BCL2 and BCL7A. These pivotal genes implicated in the pathogenesis of DLBCL stand out for their significant roles in tumor development and progression. 

The TP53 gene is over-expressed in patients’ group I, this gene is a crucial tumor suppressor gene known for its role in maintaining genomic stability by regulating the cell cycle and initiating apoptosis in response to DNA damage [32]. Mutations in TP53 are frequently observed in DLBCL, leading to a loss of function that allows neoplastic cells to escape apoptosis. This dysfunction contributes not only to the survival of genetically unstable cells but also to the accumulation of further genetic aberrations, which can drive tumorigenesis. The correlation between TP53 mutations and poor prognosis in DLBCL emphasizes the need for targeted therapies aimed at restoring p53 function or mimicking its pro-apoptotic effects [33]. 

The BCL2 gene, a master regulator of apoptosis, is often found to be overexpressed in DLBCL and is more particularly over-expressed in patients’ group II. The dysregulation of BCL2 expression enables cancer cells to evade programmed cell death, a hallmark of many malignancies. Overexpression of BCL2 can occur through various mechanisms, including chromosomal translocations, such as t(14;18), which juxtaposes the BCL2 gene to the immunoglobulin locus. This aberrant expression is associated with a poor prognosis in DLBCL patients, as it contributes to the aggressive nature of the disease. Therapeutic strategies targeting BCL2, such as BH3 mimetics, have shown promise in preclinical and clinical settings, suggesting that modulation of apoptosis pathways could be a viable approach for treating DLBCL despite various escape mechanisms [34]. 

**Figure 3:**
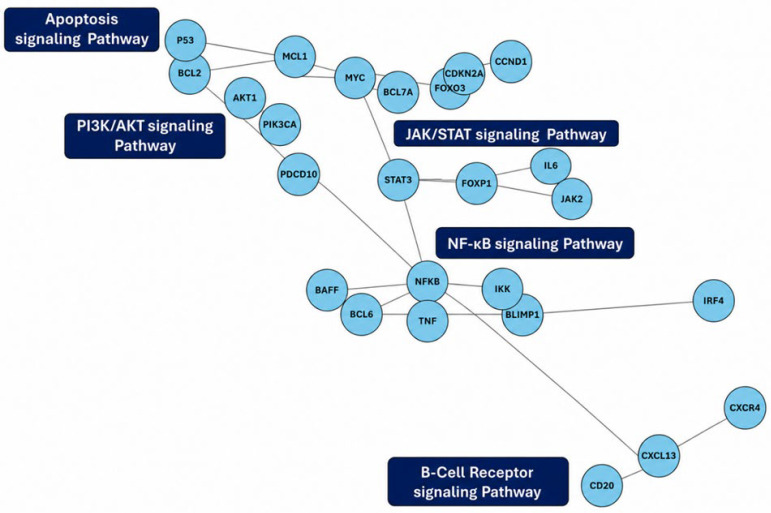
Pathway Diagram of Key Genes differentially expressed between Group I and Group II and their interactors. It illustrates the main molecular signaling pathways implicated in B-cell lymphomagenesis and survival, highlighting their interconnections and regulatory crosstalk. The diagram integrates key nodes involved in apoptosis (e.g., TP53, BCL2, MCL1), PI3K/AKT signaling (PIK3CA, AKT1), JAK/STAT signaling (JAK2, STAT3, IL6), NF-κB activation (TNF, NFKB, IKK, BAFF, BCL6), and B-cell receptor (BCR) signaling (CD20, CXCL13, CXCR4). Arrows indicate activating relationships between molecules and pathways, emphasizing how survival, proliferation, and immune-regulatory signals converge to support malignant B-cell persistence. Collectively, the network underscores the central role of coordinated oncogenic signaling in driving lymphoma pathobiology.

The BCL7A gene is over-expressed in patients’ group I. This gene was initially identified as part of the t(8;14) translocation in Burkitt lymphomas and plays a significant role in the pathogenesis of DLBCL. Its product is involved in chromatin remodeling, influencing transcription regulation, which is critical for maintaining normal B-cell function. Mutations or dysregulation of BCL7A have been associated with alterations in the B-cell receptor (BCR) signaling pathway, a key driver in the oncogenesis of DLBCL [35]. In the context of HIV-associated DLBCL, BCL7A may have a more prominent role. HIV infection leads to chronic immune activation and an increased likelihood of genetic instability in B-cells. This environment may promote mutations in BCL7A, exacerbating its oncogenic potential. HIV-positive patients with DLBCL often present with more aggressive disease, and the dysregulation of genes involved in chromatin remodeling, such as BCL7A, could contribute to this phenotype [36]. Studies have shown that BCL7A mutations are more frequent in HIV-positive DLBCL compared to non-HIV-associated cases, suggesting that this gene may act as a crucial link between the immunodeficiency state and lymphomagenesis. The complex network of molecular interactions in diffuse large B-cell lymphoma related to HIV infection is illustrated in Fig. 3.

Key differentially expressed genes, including BCL2, MYC, NF-κB, and TP53, are central to apoptotic and proliferative pathways, influenced by upstream regulators such as TNF, BAFF, and PDCD10. Pathways like JAK/STAT and PI3K/AKT further drive survival and immune evasion. Moreover, PI3K/AKT signaling has been involved in glioblastoma progression, a rare cancer that may occur synchronously with lymphoma [37]. The interactions involving BCL7A underscore its potential role in lymphomagenesis. Together, the diagram highlights critical targets for understanding and treating DLBCL. The selected genes differentially expressed between Patient Group I and II (summarized in Fig. 2) are central to key molecular pathways involved in the physiopathology of DLBCL, including cell proliferation pathways such as the PI3K-AKT pathway and B-cell receptor signaling pathway, as well as apoptosis regulators like BCL, BCL2L12, and BBC3/PUMA. Additionally, it highlights genes implicated in immune and inflammatory responses, such as IL1B, STAT1, and IRF7, and canonical signaling pathways including NF-κB and JAK-STAT. Notable genes such as REL, BCL7A, and TP53 underscore their role in the pathogenesis of HIV-associated lymphomas, while CARD11 and PLCG2 reflect disruptions in lymphocyte signaling pathways. The heatmap provides a comprehensive view of the molecular heterogeneity in lymphoma and highlights potential biomarkers and therapeutic targets, contributing to a deeper understanding of the molecular landscape of HIV-associated lymphomas and other related malignancies.

Previous studies have demonstrated the pivotal role of the immune microenvironment in influencing lymphomas behavior [38]. The presence of HIV alters the lymph node microenvironment significantly, leading to chronic B-cell activation and an increased risk of lymphomagenesis [39]. The high degree of gene expression dysregulation observed in our study is consistent with the known impact of HIV on B-cell proliferation and oncogene activation [40]. Reduced expression of interferon response genes and downstream regulators such as STAT1 and IRF7 has been associated with impaired antitumor immunity and poor prognosis in DLBCL. Furthermore, IRF8 dysregulation has been implicated in altered B-cell differentiation and contributes to immune escape in lymphoma. In the context of HIV infection, where chronic immune activation coexists with immunosuppression, such perturbations in immune signaling are likely exacerbated. As described by Mu et al., chronic inflammation in HIV leads to metabolic reprogramming and T cell dysfunction, potentially reshaping lymphomagenesis through altered cytokine signaling [39]. The differential activation of the “immune system development” pathway observed in our two patient groups may therefore reflect distinct levels of immune reconstitution or exhaustion in the tumor microenvironment, which could influence both tumor behavior and therapeutic responses. These immune-related transcriptomic patterns merit further investigation in larger cohorts, particularly to evaluate their prognostic and predictive value in HIV-associated DLBCL. Recent studies have increasingly emphasized the importance of genomic subtyping and the tumor microenvironment in understanding the heterogeneity and behavior of diffuse large B-cell lymphoma. These findings support our own observations of distinct transcriptomic profiles in HIV-related DLBCL subtypes, suggesting that both intrinsic genetic factors and extrinsic microenvironmental interactions contribute to the unique biology of these lymphomas.

While our findings offer valuable insights into the transcriptomic landscape of HIV-related DLBCL, they are subject to several significant limitations. Foremost is the small sample size of 12 biological specimens, which inherently restricts the generalizability and robustness of our conclusions. Moreover, the patient selection process in this study may have introduced potential biases that could affect the generalizability of the results (geographical location, treatment history, or comorbidities). This limited cohort size also precludes the ability to perform meaningful prognostic analyses or validate the existence of additional molecular subtypes. Furthermore, the technical variability introduced by using paraffin-embedded samples could have influenced RNA integrity and the resulting gene expression data. While we employed specialized RNA extraction and quality control protocols, FFPE samples are known to yield fragmented RNA, which may introduce biases in transcriptomic analysis. An independent replication cohort of 10 additional patients was tested to validate our findings; however, no conclusive results were obtained due to issues with RNA quality and sample labeling. The RNA extracted from these samples exhibited poor quality, which significantly impacted the integrity of the transcriptomic data. These technical difficulties underscore the need for improved sample preparation, RNA extraction protocols, and labeling strategies, particularly when working with FFPE samples. Future research should build upon the findings of this study by adopting a multi-dimensional approach to further unravel the complexity of HIV-associated DLBCL. Integrating proteomics with transcriptomic data could provide a deeper understanding of how gene expression translates into functional protein networks and reveal post-translational modifications critical to lymphoma pathogenesis. Proteomics could also help identify potential therapeutic targets by highlighting key proteins driving tumor growth, immune evasion, or resistance to therapy. Functional studies should be prioritized to validate the roles of key differentially expressed genes such as *BCL2*, *BCL7A*, and *TP53*, which were highlighted in this study. Advanced experimental approaches, including CRISPR-Cas9 gene editing and siRNA knockdowns, could directly assess the functional impact of these genes on lymphoma cell survival, proliferation, and apoptosis. Overexpression studies could further elucidate their oncogenic potential or contribution to disease progression. Such experiments would not only confirm the biological significance of these genes but also provide insights into their potential as therapeutic targets. Additionally, *in vivo* models, such as patient-derived xenografts (PDX), represent a critical next step for studying the biological impact of key genes on tumor growth and therapeutic responses. PDX models can mimic the heterogeneity and microenvironment of human DLBCL, offering a valuable platform to test novel treatments and validate findings in a clinically relevant context. To overcome the technical limitations encountered in this study, adopting advanced sequencing technologies such as RNA-Seq could greatly enhance the resolution of transcriptomic analyses. RNA-Seq would allow the detection of novel transcripts, isoform diversity, and rare mutations, which may play pivotal roles in the pathophysiology of HIV-associated DLBCL.

## 5 Conclusion

We conclude that preliminary results from our study suggest that HIV-related DLBCLs exhibit a distinct transcriptomic profile compared to HIV-negative lymphomas, influenced by immune contexture and cytokine dynamics in HIV-positive individuals. These findings contribute to novel insights into the pathobiology of HIV-associated DLBCL and support the rationale for personalized therapeutic strategies. While further validation is needed, especially in larger and more diverse cohorts, the observed gene expression patterns form a foundation for future investigations. The integration of clinical, molecular, and immunological data will be key to defining prognostic subgroups and optimizing therapeutic approaches. Multi-omics analysis may deepen understanding of tumor biology, leading to the identification of new biomarkers and actionable targets. Ultimately, these findings may shape future treatment paradigms, promoting precision medicine for improved outcomes in this vulnerable patient population.

## Data Availability

The data that support the findings of this study are openly available in the BioStudies database, under the ArrayExpress collection. The dataset is publicly accessible under the accession number E-MTAB-15172.

## Supplementary Materials

The supplementary material is available online at https://www.techscience.com/doi/10.32604/or.2026.076241/s1.
