# Supplementary material for: Transcriptomic Study of Diffuse Large B-Cell Lymphoma Associated with HIV Infection: Identification of Novel Molecular Subtypes
Source: Oncol Res. 2026 Jul 16;34(8):13. doi: 10.32604/or.2026.076241 (PMC13397319; doi:10.32604/or.2026.076241)
Supplement: Supplementary file 1 [file OncolRes-34-76241-s001.zip › TSP_OR_76241-s001.docx]

**8) Supplementary data**

***Figure S1 (color-blind reader version)***

***
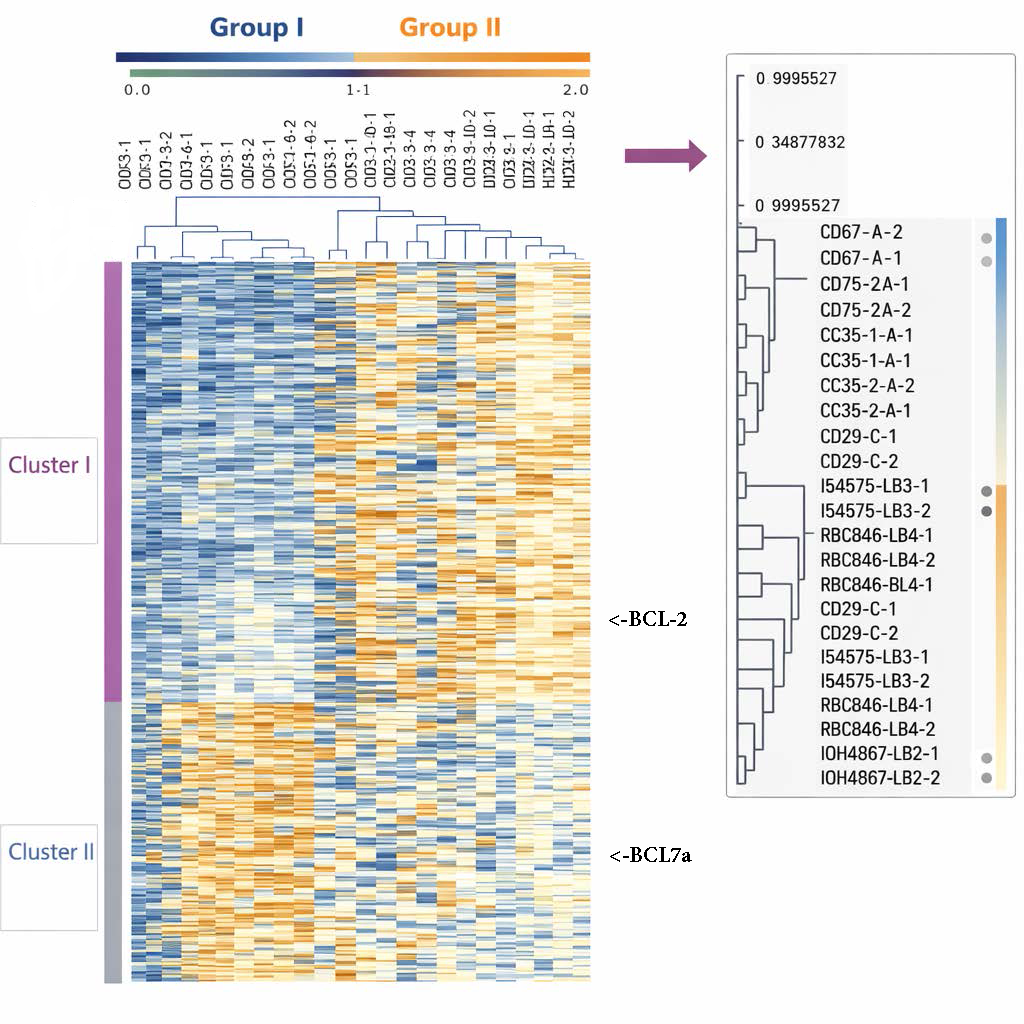
***

**Figure S1:** Transcriptomic **profiles of HIV-associated diffuse large B-cell lymphomas (DLBCL).** Heatmap representing the differential gene expression profiles of 24 samples (12 biological samples with two technical replicates each). The samples are divided into two distinct transcriptomic subgroups (Cluster I and Cluster II) based on unsupervised hierarchical clustering. Genes with significantly altered expression levels are displayed on the ordinate axis, with ***overexpressed genes shown in yellow and under expressed genes in blue***. The abscissa separates the samples into the identified clusters, highlighting distinct gene expression patterns between the groups. The clustering was performed using normalized data (quantile normalization) and median-adjusted gene expression levels, with Pearson correlation as the similarity metric and average linkage as the clustering method. These transcriptomic signatures underline the heterogeneity of HIV-associated DLBCL and suggest potential molecular differences between the two clusters.

***Figure S2 (color-blind reader version)***

******

**Figure S2: Heatmap Depicting the Expression of Differentially Expressed Genes Involved in Lymphoma Pathophysiology:** The heatmap visualizes the expression patterns of a selection of genes differentially expressed in lymphoma and hematologic malignancies, focusing on pathways critical to their physiopathology. These include key genes implicated in cell proliferation (e.g., PI3K-AKT pathway, B-cell receptor pathway), apoptosis (e.g., BCL2, BCL2L12, BBC3/PUMA), inflammation and immune signaling (e.g., IL1B, STAT1, IRF7), and canonical pathways such as NF-κB and JAK-STAT. The expression levels are median-centered to highlight ***relative upregulation (yellow) and downregulation (blue)*** across samples. Genes such as REL, BCL7A, and TP53 highlight their central role in HIV-associated lymphomas, while others like CARD11 and PLCG2 emphasize signaling disruptions in malignancies.
